# Human T leukaemia Type 1 and COVID-19

**DOI:** 10.3390/pathogens9060438

**Published:** 2020-06-03

**Authors:** Abelardo Araujo, Fabiola Martin

**Affiliations:** 1 The National Institute for Infectious Diseases, Fiocruz, Brazilian Ministry of Health and the Institute of Neurology, Federal University of Rio de Janeiro, Rio de Janeiro 21040-360, Brazil; abelardo.araujo@gmail.com; 2 Stonewall Medical Centre, Brisbane 4030, Australia; 3 School of Public Health at University of Queensland, Brisbane 4006, Australia

## Abstract

In the absence of clinical data on Human T leukaemia Type 1 and COVID-19 infection, we are providing guidance to clinicians who look after people living with HTLV-1.

Human T Leukaemia Type -1 (HTLV-1) is one of the most oncogenic human viruses [1]. It is a blood-borne virus and sexually transmitted infection (BBV/STI), causing chronic infection in humans [2]. Most people are unaware of carrying this virus, because it causes disease in about 5% of people infected [3]. An estimated 10–20 million people carry this virus worldwide [4]; therefore, about 500,000 to 1 million people suffer from HTLV-1 diseases. The most HTLV-1 common diseases are Adult T cell leukaemia (ATL) [5] and HTLV-1 associated myelopathy/Tropical spastic paraparesis (HAM/TSP) [6]. HTLV-1 chronic pulmonary disease has been reported to have detrimental effects on the health outcomes of patients [7,8]. HTLV-1 carriers and especially those suffering from HTLV-1 diseases have an impaired immune system caused by HTLV-1, which differs from HIV-1. HTLV-1 causes increased localized T cell activity and cytokine release, leading to tissue damage such as myelopathy, uveitis and broncho-alveolitis. HTLV-1 also dysregulates the CD4 and CD8 T lymphocyte proliferation, leading to a higher baseline lymphocyte count and potentially to ATL [9,10,11]. Immune-dysfunction associated with HTLV-1 is presumed to cause infective dermatitis, severe scabies, Strongyloides stercoralis hyperinfestation, and a poor tuberculin response [3,12]. 

As clinicians, what advice should we provide to our patients who are either asymptomatic HTLV-1 carriers or those who suffer from chronic inflammatory conditions such as HAM/TSP and/or ATL?

To our knowledge, there are no clinical data available on people who live with HTLV-1 and have suffered from mild or severe COVID-19 infection, but there have been several statements released on how to provide care for people who live with HIV during the COVID-19 pandemic. Reassuringly, the morbidity and mortality of people who live with HIV suffering from COVID-19 infection do not differ from people without HIV infection, if they have been sufficiently immune reconstituted while on HIV antiviral medication [13].

However, for several reasons, it will be challenging to extrapolate guidance provided for people living with HIV infection for people living with HTLV-1. There is no effective HTLV-1 antiviral therapy which can allow immune reconstitution as well as reduce tissue inflammation. People suffering from HAM/TSP often receive life-long immune-suppressive drugs such as corticosteroids, methotrexate, cyclosporine and azathioprine. People affected by ATL are already severely immunosuppressed and receive further immunosuppressive therapy such as corticosteroids, interferon, chemotherapy or myeloablation as preparation for stem-cell transplantation, and are then on life-long immunosuppressive therapy.

To date, there is no peer-reviewed publication on HTLV-1 and COVID-19 coinfection to rely on. Therefore, we make the following recommendations based on our clinical experience, scientific knowledge, recent data on risk factors for developing severe COVID-19 infection, and common sense—having worked as HTLV-1 clinical experts for an accumulated 45 years.

We recommend shielding if:

1. A person living with HTLV-1 suffers from an HTLV-1 disease, for example ATL, HAM/TSP, broncho-alveolitis or uveitis.
2. A person living with HTLV-1 suffers from other co-morbidities such as diabetes, chronic cardiac, lung, liver or kidney disease.

Shielding is when a person tries to minimize any contact with the outside world for as long as COVID-19 is endemic where they live. This means:

1. Reducing clinician appointments to a safe minimum and ideally held through telehealth.
2. Arrange for regular food and medication delivery, to avoid having to leave their home.
3. Using hand sanitation, wearing a mask and maintain a distance of 1.5–2 m from people.
4. Continue to exercise or engage in physical therapy at home on their own or supervised by a physical therapist via telehealth.
5. In a shared household, where other co-habitants access the outside world, the person living with HTLV-1 should stay in their own room as much as possible. They should use their personal towels, bathroom, and toilet if available, and arrange to use shared spaces, such as the kitchen, only when on their own.

We recommend limiting the number of persons a patient comes in contact with, implementing sensible personal distancing and wearing masks if a person living with HTLV-1 has no other co-morbidities but is

1. Male
2. Over 60 years of age
3. Have a Body Mass Index of 29.9

For all people living with HTLV-1, it is crucial to stop smoking and to keep alcohol consumption to a healthy minimum to support the immune system and to limit lung injury. It is advisable to offer eligible persons influenza and/or pneumococcal vaccination.

It is vital to rapidly seek health care advice if a person living with HTLV-1 develops symptoms associated with COVID-19, and to disclose information about their HTLV-1 infection and medication to their health care team. Ideally the patient’s HTLV physician will immediately be contacted for additional clinical input.

These recommendations differ from our routine recommendations to patients suffering from HAM/TSP, where daily exercise and stretching in- and out-doors are paramount to keep the musculoskeletal and nervous system simulated. However, we believe that sufficient stimulation can be achieved through small-dose daily exercise at home.

Furthermore, many HAM/TSP and ATL patients are wheelchair- or bed-bound, and, traditionally, some people living with HTLV-1 may sleep on the floor and not on beds, which makes mobilization on their own difficult. It may be difficult for these patients to self-isolate, since they have to rely on family, friends and carers for their daily needs. Whenever possible, these patients should be cared for by a limited number of people at home, ensuring that they are regularly mobilized to avoid pressure ulcers, infections and sepsis.

People in isolation need support with their physical needs and mental health. It can be daunting for people living with HTLV-1 to limit their routine exercise and human–human interactions. Establishing a healthy daily routine to support the mental health of a person living in isolation can be as important as regular healthy meals. Regular remote socialization via phone and video calls with friends and family supports well-being and daily meditation may strengthen the immune-system and reduce stress [14,15].

Finally, trying to expose the skin to mid-day sunshine by sitting by an open window, on the balcony, or in private garden can help maintain good bone health and the immune system through regular Vitamin D production [16].

We are aware that these recommendations are far from being evidence-based the way we would like to see. These limitations are because there are no data available that we are aware of on HTLV-1 and COVID-19 infection. Besides, unlike for HIV, there is no HTLV Health Topic provided by the World Health Organisation so that updates could be offered regularly [17]. Furthermore, unlike HIV, there is no international database to collect clinical outcome data on people with HTLV-1 and COVID-19 coinfection.

Nevertheless, we hope this information can be of use and provide clinicians with proactive actions they can take to make sure people living with HTLV-1 are safe from catching COVID-19, while maintaining good physical and mental health. 
